# Bioactive Compound, Antioxidant, and Radical Scavenging Activity of Some Plant Aqueous Extracts for Enhancing Shelf Life of Cold-Stored Rabbit Meat

**DOI:** 10.3390/antiox11061056

**Published:** 2022-05-26

**Authors:** Huda Abdalrahman Al Jumayi, Ayman Younes Allam, Alaa El-Dein El-Beltagy, Eman Hassan Algarni, Samy F. Mahmoud, Amin Abd El Halim Kandil

**Affiliations:** 1 Department of Food Science and Nutrition, College of Science, Taif University, P.O. Box 11099, Taif 21944, Saudi Arabia; huda.a@tu.edu.sa (H.A.A.J.); eman1400@tu.edu.sa (E.H.A.); 2 Department of Food Science and Technology, Faculty of Agriculture, Menoufia University, Shibin El Kom 32511, Egypt; ameen@uccd.menofia.edu.eg; 3 Department of Biotechnology, College of Science, Taif University, P.O. Box 11099, Taif 21944, Saudi Arabia; s.farouk@tu.edu.sa

**Keywords:** radical scavenging, sensory characteristic, phenolics, flavonoids, rabbit meat, natural antioxidant

## Abstract

The potential radical scavenging, antioxidant activities (DPPH and ABTS) and bioactive constituents of several plant aqueous extracts (*Curcuma longa*, CL; *Myristica fragrans*, MF; *Zingiber officinale*, ZO; *Cymbopogon citratus*, CC and *Thymus vulgaris*, TV as well as their mixture) were investigated. The effect of these extracts on quality aspects (sensory characteristic, color traits, and Thiobarbituric acid) of rabbit meat during a 16-day cold (4 ± 2 °C) storage were investigated. Total phenolics and flavonoid contents of all extracts ranged from 13.27 ± 0.57 to 25.23 ± 0.49 mg GAE/g and 6.57 ± 0.22 to 13.24 ± 0.19 mg quercetin/g, respectively. The aqueous extract of MF had the highest (*p* ≤ 0.05) ABTS scavenging activity (4.55 μ mol Te/g dry extract), whereas the highest (*p* < 0.05) DPPH scavenging activity was detected in ZO extract (9.32 μ mol Te/g dry extract). Identification of extracts’ bioactive compounds by GC-MS revealed that Eugenol (34.51%), Cinnamaldehyde (44.71%), Carvacrol (40.49%), Eicosane aldehyde (31.73%), and thymol (50.04%) are the first abundant bioactive compounds of CL, MF, ZO, CC, and TV aqueous extracts, respectively. Generally, the thiobarbituric acid reactive substances (TBARS) of all cold stored rabbit meat increased (*p* < 0.05) by increasing the storage time. The lowest TBARS values were detected for the samples treated with 0.2% of plant extracts mixture, which increased the shelf life of cold-stored rabbits by 50%. Significant (*p* < 0.05) increases in both L* and b* were observed with extended storage time. Meanwhile, the redness of the cold stored rabbit meat had an opposite trend. Treating the cold stored rabbit meat with 0.2% of the extract’s mixture doubled the storage time with acceptable odor and taste. The results indicated that the studied plant extracts may be effective against rancidity and may be used as a natural antioxidant to prolong the shelf life of cold-stored rabbit meat.

## 1. Introduction

Lipid oxidation is one of the most serious problems that decrease the shelf life of meat. Antioxidants are often used to retard lipid deterioration by scavenging the produced free radicals [1]. Some of the most used synthetic antioxidants (BHT, propyl gallate, and butyl hydroquinone) have been linked to liver damage and carcinogenesis. The potential activity of some natural antioxidants was comparable to synthetic antioxidants [2,3,4]. As a result, the need for more potent and significant plant-based natural and safe antioxidants is rising [5]. Recently, medicinal plant, herb, and spice extracts are emerging as alternatives to traditional natural preservatives, as they are generally healthy for humans and environmentally friendly [6,7,8]. Besides increasing shelf life, certain natural bioactive ingredients are useful as preventive medicines against diseases. For these reasons, various spice extracts are considered to have an important role as natural additives and preservatives for food products. The rhizome of *Curcuma longa* L. (Zingiberaceae) is often used as a preservative, flavoring, and food coloring agent worldwide due to its two major bioactive groups: phenolic curcuminoids and essential oil [9,10,11]. Along with its medicinal properties, the ginger rhizome is frequently used as a food flavoring agent or spice to enhance the taste, flavor, and oxidation stability of food [8,10,11]. Lemongrass extract is probably a high source of phenolic compounds and an effective agent for preserving unsaturated food oils [12]. Thyme extracts can be used as a raw material for polyphenols due to their high antioxidant activity, demonstrating their broad application spectrum [13]. Rabbit meat is the most promising household animal to bridge the protein gap in developing countries. Little emphasis has been focused on generating highly perishable rabbit meat products, owing to their acceptable pH value, which is within the growth range of many microbial species, and their lipids, which are highly sensitive to oxidation [11,14]. Furthermore, the scientific literature lacks information on the nutrient content and bioactivity of natural extract preservatives and their effect on the overall quality of chilled rabbit meat. The current study’s main objective was to identify the bioactive compound and radical scavenging activities of different plant aqueous extracts (*Curcuma longa*, *Myristica fragrans*, *Zingiber officinale*, *Cymbopogon citratus*, and *Thymus vulgaris*) and evaluate its efficacy as a rabbit meat natural preservative. The effects of previous extracts on quality characteristics of cooled stored rabbit meat (color, sensory attributes, and thiobarbituric acid) were evaluated.

## 2. Materials and Methods

### 2.1. Materials

Sun-dried spices of *Curcuma longa* L. (Zingiberaceae) (CL), Myristica, *Myristica* *fragrans*, (MF), Ginger *Zingiber officinale* (ZO), Lemongrass *Cymbopogon citratus* (CC), and Thyme (*Thymus vulgaris*)-(TV) were purchased from a local market’s spices shop in Shebin El-Kom, Menoufia gov.

### 2.2. Chemicals and Reagents

Folin–Ciocalteu’s reagent (FCR), sodium hydroxide (NaOH), trichloroacetic acid (TCA), gallic acid (GA), Trolox,1,1-diphenyl-2-picrylhydrazyl (DPPH), thiobarbituric acid (TBA), 2,2-azinobis (3-ethylbenzothiazoline-6-sulfonic acid) (ABTS), quercetin, and butylated hydroxytoluene (BHT) were procured from Sigma-Aldrich (St. Louis, MO, USA).

### 2.3. Preparation of Aqueous Plant Extracts

The dried spices were ground using a blender mill (Model 3510, Jenway Technology, Milano, Lombardy, Italy) and sieved 3 times using a sieve shaker from Endecott’s Limited (vibratory vertical: Octa-gon 200/Octagon 200CL/Minor 200/Air Sizer 200, London, UK) for 30 min. The process of preparing plant extracts was carried out through several stages, starting with soaking fifty grams of each plant powder in 1 L of water (*w*/*v*), and the soaking process continued in a closed conical flask for 24–36 h at room temperature using an overhead stirrer (Lab Stirrer MS-280, Misung Co., Ltd., Seoul, Korea). This stage was repeated 3 times, then the extracts were collected for each plant material separately to prepare them for the second stage, which is filtration in two successive steps; the first used three cheesecloth layers to get rid of large particles, and then the second used pre-filtration through Whatman No. 1. This was followed by the third stage, which is the concentration of the filtered extracts using a rotary evaporator under reduced pressure in a water bath at 45 °C (Rotavapor RE121, Büchi, Fawil, Switzerland). Then, the concentrated extracts are disposed through solvent by lyophilizer (2.787 sqM- Mill rock Technology, Kieffer Lane, Kingston, NY, USA) and the residue weighed, and the extraction yield for each plant material calculated. The dried powdered extract of the plant is then stored at a temperature of −20 °C. Finally, the extracts are dissolved in water at a 1:100 (*w*/*v*) ratio for further study. Finally, each plant extract powder is stored at −20 °C.

#### 2.3.1. Determination of Total Phenolic, Total Flavonoids, and Antioxidant Activities

##### Total Phenolics Content

The total phenolics content (TPC) was evaluated using the Folin-Ciocalteau method [15]. Twenty microliters of the extract were mixed with 100 μL of Folin–Ciocalteu reagent, followed by 300 μL of Na_2_CO_3_ solution (7.5%). The solutions were left at room temperature (22 ± 2 °C) for 5 min, after which 100 mL of 50% Folin-Ciocalteau phenol reagent was added and vortexed well. After incubation for 30 min, the absorbance was measured by spectrophotometer at 760 nm (SCHOTT Instruments, UviLine 9400, Mainz, Rhineland-Palatinate state, Germany). The TPC was expressed as mg gallic acid equivalents (GAEs) per g dried plant.

##### Total Flavonoids Content

The total flavonoid content (TFC) was measured using the method of [16]. Next, 0.5 mL of extract was placed in a 10 mL volumetric flask. Distilled water was added to make an even volume of 5 mL, followed by 0.3 mL NaNO_2_ (1:20); 5 min later, 3 mL AlCl_3_ (1:10) were added. After 6 min, 2 mL of NaOH (4%) were added, and then, using distilled water, the total volume increased to 10 mL. The solution was then completely mixed, and a spectrophotometer (SCHOOT instrument, UV line 9400, EU) was used to measure the absorbance at 510 nm compared to a blank. The total flavonoid content was expressed as mg quercetin equivalents (QEs) per g dried plant.

##### Identification and Quantification of Active Compounds by GC-MS

Both chromatographic tests were carried out on an Agilent system 6890-N gas chromatograph, an Agilent 7683 mass spectrometer detector [17]. A capillary column of HP-5Ms (30.0 m × 0.25 mm × 0.25 μm) was used. The carrier gas with a flow rate of 1.0 mL/min was helium (99.999%), the injection volume was 1 μL, with a 1:1 split ratio. Temperatures were kept at 100 °C for 3 min until being raised to 250 °C in 20 °C min-l increments. The Mass Spectra Library of the United States (NIST) was also used to source information. Both studies were carried out in triplicate.

##### DPPH

A DPPH radical assay was used to measure free radical scavenging activity, according to the method reported by [18]. The stock reagent solution (6 × 10^−5^ M) was prepared by dissolving 0.0024 gm of DPPH in 100 mL of Methanol and stored at −20 ± 1 °C until use. The reaction mixture was vortexed well before being kept in the dark for 30 min at room temperature. A spectrophotometer was used to measure the absorbance of the solution (6 × 10^−5^ M) at 517 nm (SCHOTT Instruments, UviLine 9400, EU). A control with no added extract was also prepared and measured. Scavenging activity was calculated as follows:

$$\mathrm{Radical}\;\mathrm{scaveging}\;\mathrm{activity}-\mathrm{DPPH}\;\left(\%\right)=\frac{{\mathrm{AC}(O)}_{517}-{\mathrm{AA}(t)}_{517}}{{\mathrm{AC}(O)}_{517}}\times 100$$

where AC(O)_517_ is the absorbance of the control at t = 0 min and AA(t)_517_ is the absorbance of the antioxidant at t = 1 h.

##### ABTS+

Based on the method of [19] with some improvements, the radical scavenging activity (ABTS+) was used to test the ability to search for ABTS+ free radicals, whereas the root cations were prepared (ABTS+) by reaction with 2.45 mm potassium sulfate (1:1 *v/v*) into a stock solution of 7.4 mM ABTS, and then the mixture was kept at room temperature overnight (12–16 h). The ABTS radical solution was diluted to absorb 1.0–1.2 with distilled water. For 60 min, an aliquot of 1 mL of the diluted spice sample was added to 50 mL of each of the above-prepared radical solutions and held in the dark. A spectrophotometer adjusted to 734 nm (SCHOOT instrument, UV line 9400, EU) was used to measure absorbance. The data is presented in the form of mean values with ±standard deviations. The extract concentration providing 0.5 of absorbance (EC_50_) was calculated from the graph of absorbance at 700 nm against extract concentration. Butylated hydroxy anisole (BHA) and -tocopherol were used as standards.

### 2.4. Quality Attribute of Cold Stored Rabbit Meat Formulated with Plant Extracts

#### 2.4.1. Preparation and Cold Storage of Formulated Rabbit Meat

Five Baladi white rabbits (2.10–2.50 kg/live rabbit) were obtained from the Faculty of Agriculture farm (Shebin El-Kom, Egypt) and slaughtered (in the same frame slaughterhouse), cleaned, deboned, and cut into small pieces following standard commercial procedures, and immediately transferred (within 10 min) in Ice boxes to the Laboratory of Meat Products, Food Science and Technology Department. The rabbit’s meat was divided into eight portions; then, the aqueous extracts (*Cymbopogon citratus*, CC; *Curcuma longa* L., CL; *Myristica fragrans*, MF; *Zingiber officinale*, ZO; *Thymus vulgaris*, TV and Mixture, equal portion of the 5th extracts, of all extracts) were added (0.2 gm/100 gm rabbit meat) [20]. The rabbit meat portion, without adding antioxidant (negative control, NC) and Positive Control (PC) prepared by adding 0.2 gm of BHT/100 gm sample were also prepared in the same manner.

Each recipe was inserted into 22 mm cellophane, sealed using a manual stuffer type cannon (SIEMSEN LTDA, CFMN model ES-08, Brusque, SC, Brazil) and promptly stored for 16 days in a refrigerator at 4 ± 2 °C for further analysis.

#### 2.4.2. Instrumental Color

Instrumental color analysis of cold stored rabbit meat samples was conducted using a scale color spectrophotometer with CIE Lab colorimeter (Hunter, Lab Scan XE, Reston, VA, USA) according to method [21]. The color was measured as CIE values (L*, a*, and b*). It was determined that the color parameters would be as follows: L* (values range from black to white for lightness), a* (values ranging from-green to redness), and b* (values ranging from–blueness to yellowness). Color difference (ΔE*) between control and cold stored rabbit meat samples was calculated from the following equation:

$$\Delta E\;=\sqrt{\Delta {L*}^{2}+\Delta {a*}^{2}+\Delta {b*}^{2}}$$

where ΔL* is the brightness difference, Δa* is the redness difference, and Δb* is the yellowness difference.

#### 2.4.3. Thiobarbituric Acid Reactive Substances (TBARS)

The TBARS (thiobarbituric acid reactive substances) distillation method was used to measure secondary lipid oxidation products, represented as malondialdehyde (MDA) equivalents. The TBARS indices were determined in triplicate samples using [22] extraction method, with Kandil, Aly-Aldin [23] modifications. The TBARS results were stated as mg of malonaldehyde/kg of rabbit meat samples and evaluated from the standard curve of TEP (1,1,3,3-tetraethyoxypropane) standards.

#### 2.4.4. Sensory Evaluation

A trained panel of 20 members (aged 21–40 years) from the Department of Food Science and Technology, Faculty of Agriculture, Menoufia University, performed the quantitative descriptive analysis (QDA) to assess the sensory quality of cooked rabbit meat (microwave oven for 6 min). Panelists were selected based on their interests and availability. The panelists were asked to rate the cooked samples taste and aroma/odor using a scale point ranging from 0 to 10, were 10 = excellent; 9 = Very good; 8 = Good; 7 = Acceptable; 6 = poor. The product has been defined as unacceptable after the onset of a bad odor or unpleasant taste [23].

### 2.5. Statistical Analysis

Data were analyzed using the statistical package SAS version 9.4 (2013). The study was replicated three times, and two measurements were conducted per replicate. Data were analyzed using the SPSS program. Results were expressed as the mean ± SD. Data for multiple variable comparisons were analyzed by analysis of variance (ANOVA). Mean values of different parameters were used to compare DPPH, full phenolics, hunter color values, and sensory characteristics. The means were separated with the least significant difference (LSD) procedure. Significance between groups was conducted using Duncan’s analysis. The statistical significance was identified at the 95% confidence level (*p* < 0.05).

## 3. Result

### 3.1. Extraction Yield, Total Phenolic, Total Flavonoids, and Antioxidant Capacity

The extraction yield of the plant aqueous extracts ranged from 2.62 ± 0.56% to 2.76 ± 0.42% (Table 1). The Curcuma longa L. extract had the highest yield (*p* ≤ 0.05), while the lowest yield (*p* <0.05) was recorded for Thymus vulgaris (2.23 ± 0.35). The highest (*p* ≤ 0.05) DPPH scavenging activity was observed in Z. officinale aqueous extract (9.22 ± 0.17 μ mol TE/g), the other extracts could arrange as the following decreasing order M. fragrans (8.44 ± 0.14 μ mol TE/g), C. longa (7.23 ± 0.18 μ mol TE/g), C. citratus (5.44 ± 0.12 μ mol TE/g) and T. vulgaris (3.87 ± 0.11 μ mol TE/g). The ABTS + antioxidant activity results showed a similar trend of the DPPH with slightly lower values (Table 1). The radical reduction of ABTS+ by antioxidants ranged between 1.89 and 4.12μ mol TE/g. *M. fragrans* (4.55 ± 0.11) had the highest (*p* < 0.05) antioxidant activity; Z. officinale (3.48 ± 0.09) and C. longa (3.25 ± 0.12), respectively, had the lowest antioxidant activity in *T. vulgaris* and *C. citratus* (2.11 ± 0.09 and 1.89 ± 0.11, respectively). Extracts of *Z. officinale*, *M. fragrans*, *C. longa*, *T. vulgaris*, and *C. citratus* contained 13.24 ± 0.19, 11.25 ± 0.22, 10.24 ± 0.19, 7.21 ± 0.21, and 6.57 ± 0.22 mg QE/ g sample of flavonoid, respectively.

### 3.2. Identification and Characterization of Bioactive Compound

The bioactive components of the investigated extracts were identified by GC-MS (Table 2, Table 3, Table 4, Table 5 and Table 6). Eugenol (34.51%) appeared as the primary ingredient (Table 2) of *Curcuma longa* L. aqueous extract, followed by Turmerone (21.73%) and ar-turmerone (18.10%), while Ar-Curcumene was the lowest (1.50%). Cinnamaldehyde, γ-terpinene, and 4-Allyl-2-methoxy phenol (44.71%, 19.37%, and 18.09%, respectively) were the abundant bioactive compounds of *Myristica fragrans* (Table 3). Cinnamaldehyde, the abundant compound of the *Myristica fragrans* aqueous extract, might be responsible for its radical scavenging activity [11]. Carvacrol, thymol, Methylheptenone, and Tricyclene (40.49%, 19.02%, 7.66%, and 6.95%, respectively) were the main compounds in *Zingiber officinale* extract (Table 4). Eicosane aldehyde, caryophyllene, octadecanoic acid, and hexadecenoic acid (31.73%, 11.22%, 7.10%, and 6.85, respectively) were the main compounds in Cymbopogon citratus extract (Table 5) and were responsible for his activities as an antimicrobial and antioxidant. The main component of Thymus vulgaris (Table 6) were thymol, p-Cymene, and γ-Terpineol (50.04%, 20.82, and 18.80%, respectively).

### 3.3. Quality Attributes of Cold-Stored Rabbit Meat Formulated with Aqueous Plant Extracts

#### 3.3.1. Color Values

**Figure 1:**
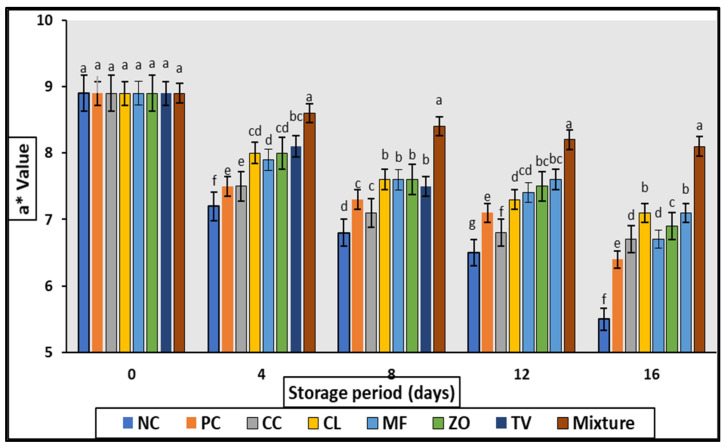
Changes in a* value of cold stored rabbit meat formulated with different aqueous plant extracts. Columns with different letters are significantly different (*p* < 0.05).

**Figure 2:**
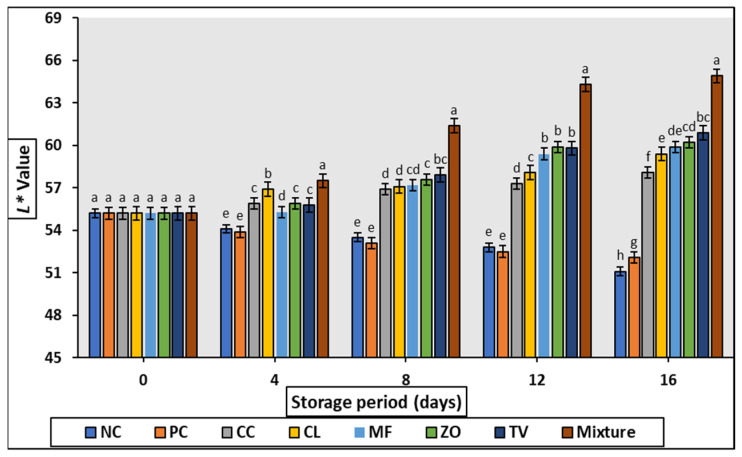
Changes in L* value of cold stored rabbit meat formulated with different aqueous plant extracts. Columns with different letters are significantly different (*p* < 0.05).

**Figure 3:**
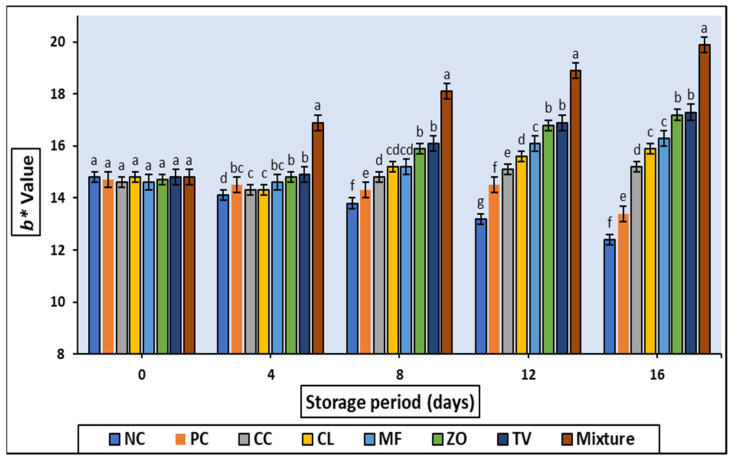
Changes in b* value of cold stored rabbit meat formulated with different aqueous plant extracts. Columns with different letters are significantly different (*p* < 0.05).

**Figure 4:**
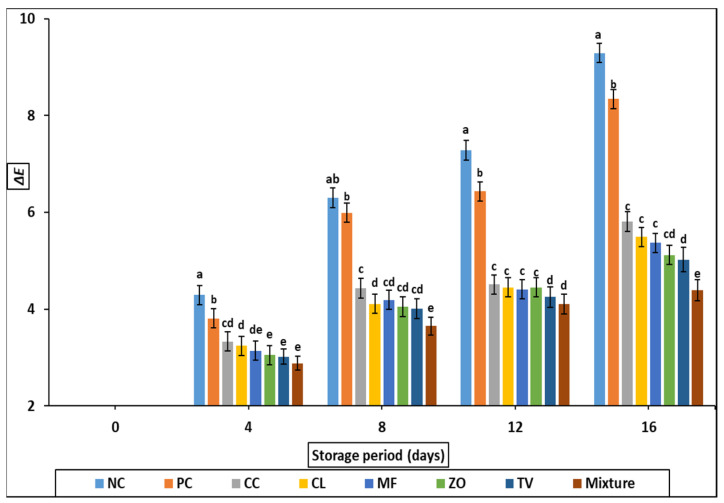
Color difference changes (∆E*) of cold stored rabbit meat formulated with different aqueous plant extracts. Columns with different letters are significantly different (*p* < 0.05).

The CIE values of raw rabbit meat samples formulated with 0.2% of different aqueous extracts are illustrated in Figure 1, Figure 2, Figure 3 and Figure 4. Generally, no significant differences (*p* > 0.05) were observed among all samples (including control) at 0 days of storage, while gradual increase (*p* < 0.05) of lightness (L*) values were noticed with increasing storage period compared with control samples.

The rabbit meat samples treated with aqueous plant extracts showed a higher (*p* ≤ 0.05) red color, and therefore higher values for a* meanwhile, a gradual decrease (*p* < 0.05) in redness was noticed with a prolonged storage period (Figure 2).

The samples treated with ZO, TV, and the mixture had the highest (*p* < 0.05) yellowness (b*) value during all the storing times. Additionally, all treatments exhibited significantly (*p* < 0.05) higher b* values than the control (Figure 3). As it turns out that the color has stabilized significantly (*p* < 0.05) to include extracts resulting in such an increase (*p* < 0.05) in the yellowish color of formulated rabbit meat samples.

As the storage period increased (Figure 4), the color difference changes (∆E*) of rabbit meat samples increased (*p* < 0.05). After 8, 12, and 16 days of refrigerated storage, the color difference change of rabbit meat samples treated with C. longam, M. fragrans, and Z. officinale extracts were more prominent (*p* < 0.05) than other treated samples.

#### 3.3.2. TBARS Contents

**Figure 5:**
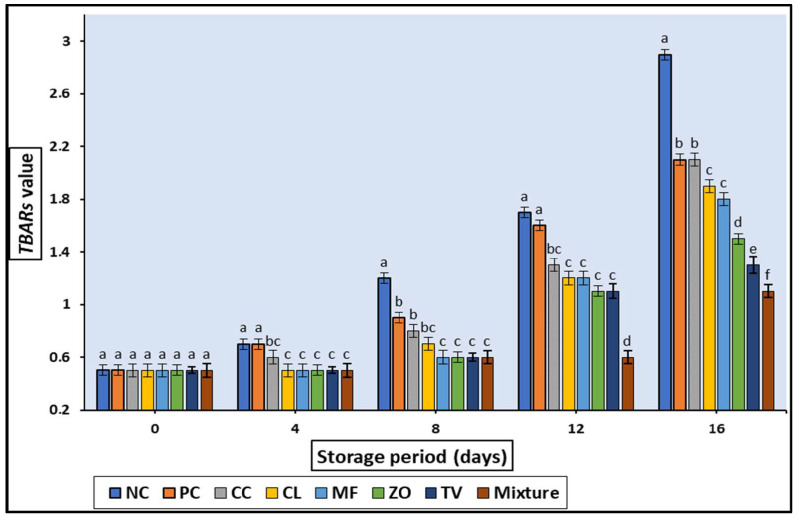
Effect of treating with different plant aqueous extracts on TBARs value of cold stored rabbit meat. Columns with different letters are significantly different (*p* < 0.05).

The effect of adding 0.2% of plant aqueous extracts on fat and lipid oxidation of raw rabbit meat stored at 4 ± 2 °C for 16 days is shown in Figure 5. At 0 days, the TBARS values of all treatments had no significant (*p* > 0.05) differences. TBARS values were significantly (*p* < 0.05) higher in NC samples during all the storage periods compared with the treated samples. Extending the storage time up to eight days increased (*p* < 0.05) the TBARS of all treatments. According to the Egyptian standard, the maximum value of TBARS in rabbit meat is 0.9 mg MDA/kg. As illustrated in our results, both negative and positive control reached the upper TBARS quality standard limit by the eighth day of storage; meanwhile, the samples treated with 0.2% of plant extracts showed TBARS values ranged from 0.6 to 0.8 mg MDA/kg, which maintain its compliance with legislation. The lowest TBARS values were detected for the samples treated with 0.2% of extracts mixture, which still comply, with up to 12 days of storage.

#### 3.3.3. Sensory Evaluation

The sensory characteristics results (taste and odor) of cooked rabbit meat formulated with different plant aqueous extracts are shown in Table 7. Generally, cooked rabbit meat samples on the initial day of storage had a significantly (*p* < 0.05) good odor and taste, whereas a significant (*p* < 0.05) decrease in both attributes was noticed by increasing the storage days in all treated samples. The untreated sampled showed a dramatic (*p* < 0.05) odor and taste deterioration by the fourth storage day and became completely unacceptable. Meanwhile, the samples formulated with 0.2% of the different studied plant extracts did well for up to four days of storage (and up to eight days for the samples treated with 0.2% of the mixture of spice extracts). By the eighth day, all untreated samples and those treated with 0.2% of all extracts became unacceptable for the panelists. The samples treated with 0.2% spices mixtures kept their acceptability until the eighth day of storage. Generally, rabbit meat is highly susceptible to lipid oxidation during cold storage in aerobic conditions [14].

## 4. Discussion

Plant extracts are rich in many important non-nutritional and biologically active compounds such as phytochemicals. Among these different phytochemicals, it was found that total phenolic (TPC) and total flavonoid compounds (TFC) have attracted attention in the last few years in various food products [24,25,26]. Many studies have demonstrated a positive relationship between TPC and antioxidant activity in several fruits and plants [5,26]. Such a relationship clearly appeared in our results, where the Zingiber officinale aqueous extract which contain the highest total flavonoid compounds (13.24 mg quercetin/g) and the second highest value of TPC (23.14 mg GAE/g) showed the highest (*p* ≤ 0.05) DPPH radical scavenging activity and the second highest value of ABTS activity. Meanwhile, Cymbopogon citratus water extract, which had the lowest (*p* ≤ 0.05) TVC, showed the lowest ABTS scavenging activity.

Gas chromatography–Mass (GC-MS) identification of the bioactive compound of the studied plant extract declared that two abundant compounds represent more than 50% of the bioactive compound in every extract. These compounds are Eugenol and Turmerone (56.6%) in *Curcuma longa* L; Cinnamaldehyde and γ-terpinene (64.08%) in *Myristica fragrans*; Carvacrol and thymol (59.51%) *Zingiber officinale*; Eicosane aldehyde and caryophyllene (42.95%) in *Cymbopogon citratus* extract; thymol and p-Cymene (70.86%) in *Thymus vulgaris*. The antioxidant activity of these compounds is believed to be due to the high potential of oxidation, which allows them to work as agent’s reduction, donor’s hydrogen, and oxygen single quenchers [27]. Eugenol had an antioxidant effect to inhibit lipid peroxidation, which is potent, to form complexes with reduced metals [28]. Ogata et al. [29] studied the lipid peroxidation inhibitory mechanism of eugenol and stated that eugenol might be trapping the active oxygen and interfering with the chain reactions. Eugenol has the highest antioxidant activity compared to the other essential oil components [30]. Previous studies reported that extracts of *C. longa*, *M. fragrans*, and *Z. officinale* were considered high powerful natural antioxidants. This indicates that the strong activity of *C. longa* and *M. fragrans* may be due to the presence of eugenol and thymol, as it is the main component of *C. longa* and *M. fragrans*, which are known for their high antioxidant activity [28,29,30]. Also, *Zingiber officinale* was found to have high antioxidant activity, possibly due to the presence of Carvacrol. Although higher antioxidant activity contains *T. vulgaris* and *C. citratus* at a lower level of phenols, it showed less radical cleaning activity when compared to the other extracts [31].

The quality attributes (CIE color value, TBARS, sensory properties) of cold stored rabbit meat formulated with 0.2% of different aqueous plant extract are illustrated in Figure 1, Figure 2, Figure 3, Figure 4 and Figure 5 and Table 7. Color is one of the most important characteristics that affects the appearance, presentation, and acceptability. The redness (a*) of the stored samples tends to decrease with increasing storage time. This tendency may be attributed to interference with fat oxidation during myoglobin oxidation; this could be maintained by the TBARS values of the same samples which increased slightly over the storage period. Such decreases in a * value were detected in chopped and super chilled rabbit during 8–12 days of storage by Lan et al. However, it is known that the decrease in red intensity during storage is linked to the correlation between color oxidation and fat oxidation in meat [32,33]. Oxidation of the pigment may stimulate the oxidation of fat, lead the free radicals produced during oxidation to oxidate iron atoms, or molecules to denature myoglobin, which changes the color of negative products.

In addition to microbial spoilage, the key factor restricting the shelf life of rabbit meat is lipid oxidation and chemical degradation. Thus, the current study’s findings indicate that adding the studied extracts might retard rabbit meat lipid oxidation. Such retardation may be due to the presence of phenolic compounds which might act through different mechanisms (reducing agent, blocking free radicals, ion chelating agents, and /or reducing reactive oxygen). Abdelmaguid et al. [14] observed similar results and cleared that treating rabbit meat with olive leaf extract could extend the shelf life of cold-stored rabbit meat up to nine days. Therefore, many researchers confirmed these findings and applied different natural antioxidant sources to reduce lipid oxidation in cooked meats during cold storage [34,35].

Taste and odor of rabbit meat were equally represented sensitive sensory characteristics. The dramatic deterioration of control samples might be due to odor production due to lipid oxidation, microbial growth, and/or ammonia production from protein breakdown. According to [14,36], the development of chilled stored rabbit meat’s putrefactive odor might come from protein decomposition by microbial enzymes, which produce some off-odor compounds (hydrogen sulfide, ammonia, and mercaptan).

## 5. Conclusions

Natural antioxidants are being increasingly exploited on a global scale due to their safety and nutritional benefits, of which their phytochemical contents are a valuable component. Considering their adequate phenolic and flavonoid content, bioactive compounds, as well as antioxidant activities, aqueous extracts can be convenient to use as alternatives to the synthetic antioxidants. The combining of plant extracts has an increased ability to depress lipid oxidation in chilled rabbit meat products, significantly preserving organoleptic properties and extending shelf life. The addition, the spice extracts reduced the TBARS and kept the sensory characteristics of treated rabbit carcasses longer.

## Figures and Tables

**Table 1:** Extraction yield, phenolic, flavonoids content, and antioxidant activities of some aqueous plant extracts.

Activity	*Curcuma longa* L.	*Myristica fragrans*	*Zingiber officinale*	*Cymbopogon citratus*	*Thymus vulgaris*
Extraction yield (%)	2.84 ± 0.47 ^a^	2.64 ± 0.44 ^c^	2.42 ± 0.38 ^d^	2.74 ± 0.41 ^b^	2.23 ± 0.35 ^e^
DPPH (μ mol TE/g)	7.23 ± 0.18 ^c^	8.44 ± 0.14 ^b^	9.22 ± 0.17 ^a^	5.44 ± 0.12 ^d^	3.87 ± 0.11 ^e^
ABTS (μ mol TE/g)	3.25 ± 0.12 ^c^	4.55 ± 0.15 ^a^	3.48 ± 0.09 ^b^	1.89 ± 0.11 ^e^	2.11 ± 0.09 ^d^
Total phenolic content (mg GAE/g)	25.23 ± 0.49 ^a^	21.25 ± 0.55 ^c^	23.14 ± 0.4 5 ^b^	16.24 ± 0.48 ^d^	13.27 ± 0.57 ^e^
Total flavonoid content (mg quercetin/g)	10.24 ± 0.19 ^c^	11.25 ± 0.22 ^b^	13.24 ± 0.19 ^a^	6.57 ± 0.22 ^e^	7.21 ± 0.21 ^d^

Duncan’s multiple tests show that means followed by different small letters in the same raw are substantially different (*p* < 0.05).

**Table 2:** Identification of *Curcuma longa* L. aqueous extract bioactive compounds.

Components	Retention Time (min)	Composition (%)
Eugenol	22.36	34.51
(E)-Caryophyllene	26.51	1.68
Ar-Curcumene	28.14	1.5
α--Zingiberene	29.31	2.34
β--Sesquiphellandrene	30.2	2.42
ar-turmerone	33.24	18.1
Turmerone	34.65	21.73
Curlone	34.65	13.76
unidentified	15.6	3.05

**Table 3:** Identification of *Myristica fragrans* aqueous extract bioactive compounds.

Component	Retention Time (min)	Composition (%)
Cinnamaldehyde	20.65	44.71
γ-terpinene	19.10	19.37
4-Allyl-2-methoxyphenol (eugenol)	14.25	18.09
α-pinene	6.25	9.72
myristicin	6.18	0.79
β-pinene	4.52	0.19
sylvestrene	4.77	1.01
elemicin	4.32	3.76
safrole	4.22	0.17
α-terpinyl acetate	34.01	1.17
eucalyptol	2364	1.00

**Table 4:** Identification of Zingiber officinale aqueous extract bioactive compounds.

Component	Retention Time (min)	Composition (%)
Carvacrol	22.45	40.49
Thymol	23.17	19.02
β-Phellandrene	7.48	0.59
Methylheptenone	7.12	7.66
Camphene	5.78	3.87
2-Heptanone	4.41	0.20
Tricyclene	5.23	6.95
Borneol	11.87	1.28
α-Pinene	5.47	2.03
2,3-epoxygerianial	14.81	0.14
Neral	15.89	3.42
trans-β-bergamotene	22.17	0.42
α-Farnesene	22.14	6.89
(E)-γ-Bisabolene	24.89	0.44
Sesquisabinene	24.17	5.28
(+)(E)-Caryophyllene	21.40	0.18
unknown	29.87	1.15

**Table 5:** Identification of *Cymbopogon citratus* aqueous extract bioactive compounds.

Component	Retention Time (min)	Composition (%)
Hexadecanoic acid (Palmitic)	16.46	6.85
Hepta-9,10,11-trienoic acid	18.17	14.72
Octadecanoic acid (Stearic acid)	18.31	7.10
2-ethenyltetradecan-1-ol	19.26	11.22
Eicosane aldehyde	20.77	31.73
1-ethoxyoctadecane	20.91	12.84
Caryophyllene	23.54	11.22
Humulene 25.78 3.5	23.75	2.92
(-)-terpinen-4-ol	10.21	0.20
Linalool	6.89	0.33
neryl butyrate	5.98	0.66
Unidentified	23.22	0.21

**Table 6:** Identification of Thymus vulgaris aqueous extract bioactive compounds.

Component	Retention Time (min)	Composition (%)
Thymol	1.17	50.04
p-Cymene	1.48	20.82
α-Thujene	4.58	0.60
α-Pinene	7.84	0.41
Camphene	4.15	0.41
Vinyl amyl carbinol	2.14	1.09
Myrcene	22.41	2.76
2-Ethylhexanol	1.24	0.27
α-Terpinene	9.47	2.09
Limonene	4.24	0.64
γ-Terpineol	1.24	18.80
Trans-p-menth-2-en-1-ol	9.34	0.54
Linalool	8.54	0.25
α-Humulene	1.23	0.19
Caryophyllene oxide	1.28	1.09

**Table 7:** Sensory evaluation of cold stored rabbit meat formulated with some plant aqueous extracts.

Sensory Properties (Odor and Taste)												
Treatments	Odor					L.S.D.	Taste					L.S.D.
	0	4	8	12	16		0	4	8	12	16	
PC	9.3 ± 0.15 ^Aa^	6.6 ± 0.41 ^Ec^	3.6 ± 0.45 ^Cc^	Not	Not	0.52	9.2 ± 0.35 ^Aa^	7.2 ± 0.31 ^Bc^	4.2 ± 0.38 ^Cc^	Not	Not	0.44
NC	9.2 ± 0.13 ^Aa^	6.6 ± 0.42 ^Ec^	3.6 ± 0.41 ^Cc^	Not	Not	0.52	9.2 ± 0.33 ^Aa^	7.2 ± 0.35 ^Bc^	4.2 ± 0.35 ^Cc^	Not	Not	0.44
CC	9.3 ± 0.12 ^Aa^	7.6 ± 0.33 ^Bb^	5.2 ± 0.52 ^Cb^	3.8 ± 0.54 ^Db^	2.6 ± 0.22 ^Ec^	0.72	9.2 ± 0.31 ^Aa^	8.2 ± 0.38 ^Bb^	6.2 ± 0.31 ^Cb^	4.5 ± 0.34 ^Dc^	3.8 ± 0.31 ^Db^	0.80
CL	9.3 ± 0.22 ^Aa^	7.7 ± 0.24 ^Bb^	5.3 ± 0.54 ^Cb^	4.0 ± 0.42 ^Db^	2.5 ± 0.45 ^Ec^	0.81	9.1 ± 0.29 ^Aa^	8.3 ± 0.36 ^Ab^	6.1 ± 0.39 ^Bb^	4.9 ± 0.39 ^Cb^	3.8 ± 0.36 ^Db^	0.91
MF	9.3 ± 0.25 ^Aa^	7.5 ± 0.36 ^Bb^	5.2 ± 0.52 ^Cb^	4.2 ± 0.33 ^Db^	2.8 ± 0.15 ^Ec^	0.68	9.2 ± 0.25 ^Aa^	8.3 ± 0.38 ^Ab^	6.2 ± 0.33 ^Bb^	5.1 ± 0.28 ^Cb^	3.9 ± 0.39 ^Db^	0.90
ZO	9.3 ± 0.31 ^Aa^	7.4 ± 0.31 ^Bb^	5.3 ± 0.52 ^Cb^	4.1 ± 0.41 ^Db^	2.9 ± 0.24 ^Ec^	0.55	9.2 ± 0.41 ^Aa^	8.2 ± 0.35 ^Bb^	6.3 ± 0.34 ^Cb^	5.2 ± 0.29 ^Db^	4.0 ± 0.37 ^Eb^	0.92
TV	9.3 ± 0.22 ^Aa^	7.5 ± 0.32 ^Bb^	5.4 ± 0.56 ^Cb^	4.2 ± 0.29 ^Db^	3.9 ± 0.52 ^Eb^	0.41	9.2 ± 0.39 ^Aa^	8.5 ± 0.33 ^Aa^	6.5 ± 0.32 ^Bb^	5.1 ± 0.25 ^Cb^	4.1 ± 0.31 ^Db^	0.83
Mixture	9.3 ± 0.36 ^Aa^	8.9 ± 0.45 ^Ba^	7.6 ± 0.51 ^Ca^	6.4 ± 0.34 ^Da^	6.1 ± 0.41 ^Ea^	0.52	9.2 ± 0.35 ^Aa^	8.8 ± 0.31 ^Aa^	7.9 ± 0.31 ^Ba^	6.7 ± 0.39 ^Ca^	5.7 ± 0.30 ^Da^	0.54
L.S.D.	0.50	0.41	0.44	0.64	0.55	-	0.23	0.24	0.33	0.25	0.31	-

Nots: Means of triplicate Standard Deviation (±SD); The means accompanied by different capital letters in the same raw (effect of storage period) are significantly different (*p* < 0.05). The means followed by different small letters in the same column (effect of treatments) are significantly different (*p* < 0.05).

## Data Availability

Data contained in the article.

## Abbreviations

NC	negative control; no extract.
PC	Positive control with 0.2% BHT (butylated hydroxyl toluene).
CC	Treatment with *Cymbopogon citratus* water extract (0.2% *w*/*w*).
CL	Treatment with *Curcuma longa* L. water extract (0.2% *w*/*w*).
MF	Treatment with *Myristica fragrans* water extract (0.2% *w*/*w*).
ZO	Treatment with *Zingiber officinale* water extract (0.2% *w*/*w*).
TV	Treatment with *Thymus vulgaris* water extract (0.2% *w*/*w*).
Mixture	Treatment with an equal amount of the previous five (CC, CL, MF, ZO and TV) aqueous extracts (0.2% *w*/*w*).
